# 201 Is Time on Their Side? Real World Environmental Services Practices and the Contact Time Paradox

**DOI:** 10.1017/ash.2026.10589

**Published:** 2026-06-23

**Authors:** Peggy Douglas, Louise Lie, Malika Elkayssi, Georgia O’Neil, Amanda Pease, Anne Hamilton, Svaya Olin, Tim Taylor, Michelle McIntosh, Wesley Wang, Regina Kwon, Nandita Mani, Geoffrey Gottlieb, Seth Cohen

**Affiliations:** 1 University of Washington Medical Center; 2 UW Medicine; 3 University of Washington

## Abstract

**Background** At University of Washington Medical Center (UWMC), patients undergoing evaluation for extrapulmonary tuberculosis (EPTB) are routinely placed in airborne precautions and undergo pulmonary tuberculosis (PTB) rule-out, regardless of epidemiologic risk factors or pulmonary symptoms. This approach was implemented to support early identification of occult PTB in asymptomatic individuals. While prior small studies suggest PTB may occur infrequently among patients with EPTB without pulmonary symptoms, CDC guidance recommends PTB evaluation based on clinical suspicion and epidemiologic risk. The clinical yield and operational, financial, and environmental impacts of UWMC’s universal approach are unclear; this study characterizes its utility and burden. Methods We conducted a retrospective descriptive analysis of adult patients evaluated for EPTB at a low TB-incidence tertiary-care academic medical center (April 2024–December 2025). Data were exempt from IRB review. Outcomes included patient characteristics (including hematology-oncology status), diagnostic testing, TB identification, airborne precaution duration, and costs. EPTB evaluation was defined by initiation of the institutional pathway (e.g., airborne precautions and PTB rule-out orders). Laboratory and PPE costs were estimated using institutional unit costs. PPE utilization was estimated using a workflow-based assumption of 14 room entries per day. Sensitivity analyses used 10 and 18 daily room entry assumptions. Environmental impact was assessed descriptively via estimated PPE encounters. Results Thirty-eight patients undergoing evaluation for EPTB were included (133 airborne precaution days). Twenty-five (65%) had no identified epidemiologic TB risk factors; many had nonspecific pulmonary findings. Two (5%) had hematologic malignancies. Estimated laboratory cost was $4,640.38 ($122.12/patient). Estimated PPE cost was $2,197.16 ($57.82/patient), with sensitivity estimates ranging from $1,510–$2,719. The combined estimated direct cost was $6,837.54 ($179.94/patient). Under base-case assumptions, airborne precautions generated 1,862 PPE encounters. Among this 38-patient EPTB evaluation cohort, 1 case of EPTB and 0 cases of PTB were identified. Conclusions In this cohort, routine PTB rule-out and airborne precautions were applied during evaluation for possible EPTB, yet no patients were diagnosed with PTB. Although direct laboratory and PPE costs were modest, this practice generated substantial airborne precaution days and imposed unmeasured burdens, including delays in care/procedures, reduced quality of care, and additional staff/provider time. Environmental impacts beyond estimated PPE utilization were not captured. These findings suggest the current universal approach overestimates risk in this population. A targeted, risk-stratified approach aligned with epidemiologic risk, clinical suspicion, and guideline-based recommendations may reduce unnecessary precautions and testing while maintaining patient and staff safety.

